# A cell-cycle independent role for p21 in regulating synovial fibroblast migration in rheumatoid arthritis

**DOI:** 10.1186/ar1999

**Published:** 2006-07-17

**Authors:** James M Woods, Karolina Klosowska, Darrin J Spoden, Nataliya G Stumbo, Douglas J Paige, John C Scatizzi, Michael V Volin, Malathi S Rao, Harris Perlman

**Affiliations:** 1 Department of Microbiology and Immunology, Chicago College of Osteopathic Medicine, Midwestern University, Downers Grove, IL 60515, USA; 2 Department of Molecular Microbiology-Immunology, Saint Louis University, School of Medicine, St Louis, MO 63104, USA

## Abstract

Rheumatoid arthritis (RA) is characterized by synovial hyperplasia and destruction of cartilage and bone. The fibroblast-like synoviocyte (FLS) population is central to the development of pannus by migrating into cartilage and bone. We demonstrated previously that expression of the cell cycle inhibitor p21 is significantly reduced in RA synovial lining, particularly in the FLS. The aim of this study was to determine whether reduced expression of p21 in FLS could alter the migratory behavior of these cells. FLS were isolated from mice deficient in p21 (p21^(-/-)^) and were examined with respect to growth and migration. p21^(-/-) ^and wild-type (WT) FLS were compared with respect to migration towards chemoattractants found in RA synovial fluid in the presence and absence of cell cycle inhibitors. Restoration of p21 expression was accomplished using adenoviral infection. As anticipated from the loss of a cell cycle inhibitor, p21^(-/-) ^FLS grow more rapidly than WT FLS. In examining migration towards biologically relevant RA synovial fluid, p21^(-/-) ^FLS display a marked increase (3.1-fold; p < 0.05) in migration compared to WT cells. Moreover, this effect is independent of the cell cycle since chemical inhibitors that block the cell cycle have no effect on migration. In contrast, p21 is required to repress migration as restoration of p21 expression in p21^(-/-) ^FLS reverses this effect. Taken together, these data suggest that p21 plays a novel role in normal FLS, namely to repress migration. Loss of p21 expression that occurs in RA FLS may contribute to excessive invasion and subsequent joint destruction.

## Introduction

Proper regulation of the mammalian cell cycle is vital for cellular homeostasis. Alterations in the cell cycle components have been associated with several disease states. Progression through the different phases of the cell cycle is dependent on the activities of cyclin dependent kinases (cdks) bound to their cognate cyclins [1,2]. Another level of cell cycle regulation is affected by the cdk inhibitors, which bind to cdk or cdk-cyclin complexes and inhibit their kinase activity. The cdk inhibitors are grouped into two categories based on homology and preferential cdk-cyclin binding (Inks, comprising p15, p16, p18 and p19; and Cip/Kip, comprising p21, p27 and p57). Overexpression of any of the cdk inhibitors will induce G1-cell cycle arrest [3]. Deficiencies in p16 [4,5], p18 [6], p19 [7,8], p27 [6,9-11] and p21 [12,13] may lead to or enhance oncogenesis. However, to date, only the loss of p21 has been associated with the development of an autoimmune disease phenotype [14,15]. New unexpected roles for p21 and p27 have recently been revealed in apoptosis and transcriptional activation [16]. Moreover, p27 has been found to play a novel role in regulating cell migration, where fibroblasts lacking p27 exhibit dramatically decreased motility in comparison with controls [17].

Rheumatoid arthritis (RA) is a chronic inflammatory and destructive disease [18]. The fibroblast-like synoviocytes (FLS) that comprise the synovial lining, a thin membrane in direct contact with cartilage and bone, are one of the principal cells responsible for the pathogenesis of RA. In RA, the FLS increase in number and produce pro-inflammatory cytokines, chemokines, and matrix-metalloproteinases that promote inflammation and joint destruction. Isolated RA FLS induce arthritis when transferred to the knees of healthy SCID mice in the absence of a functional immune system [19]. Recently, the role of p21 in the pathogenesis of RA has been investigated. The expression of p21 is reduced in RA when compared to osteoarthritis synovial tissue [20], particularly in the FLS population. Overexpression of p21 inhibits proliferative and inflammatory properties in FLS isolated from patients with RA [20-24] and results in the amelioration of experimental arthritis in mice and rats [21-24]. These data demonstrate that p21 normally functions to inhibit the inflammatory response in FLS. Herein, we investigate the role of p21 in modulating the migration of FLS. Our data suggest that p21 normally represses migration in FLS and that loss of p21 expression that occurs in RA may contribute to excessive invasion by FLS.

## Materials and methods

### Mouse synovial fibroblasts

p21^(-/-) ^(B6;129S2-*Cdkn1a*^*tm1Tyj*^/J) and wild-type (WT; B6;129SF2/J) mice were purchased from the Jackson Laboratory (Bar Harbor, Maine, USA). Mouse knees were excised from WT or p21^(-/-) ^mice and pooled. Isolated mouse synovial tissues were digested with collagenase, dispase, and DNAse I, and single cell suspensions were obtained [20,25,26]. A homogenous population was determined by flow cytometry (<1% CD11b, <1% F4/80, and <1% CD45). FLS were cultured in a standard DMEM + 10% FBS (Hyclone Inc., Logan, UT, USA) with penicillin/streptomycin. FLS were used at passage ≥3, at which time cells were considered to be a more homogeneous population of fibroblasts. All mouse studies were performed with Animal Care and Use Committee approval at St Louis University.

### Patient samples

Synovial fluid (SF) specimens were collected during arthrocentesis from patients who met the American College of Rheumatology criteria for a diagnosis of RA. All specimens were obtained with approval of Midwestern University's Institutional Review Board.

### FLS proliferation

WT and p21^(-/-) ^fibroblasts were plated at 1.8 × 10^4 ^cells/well in 1 ml DMEM + 10% FBS in a standard 24-well tissue culture plate and incubated at 37°C. At different time points cells were washed with PBS, trypsinized and their number quantified by hemocytometer counts with trypan blue. Analysis was performed in triplicate wells and results are expressed as the mean ± standard error (SE).

### Chemotaxis and checkerboard assays with RA synovial fluid

Chemotaxis was performed with minor modifications to a protocol previously optimized for RA synoviocyte chemotaxis [27]. WT and p21^(-/-) ^FLS were cultured for 18 h in DMEM containing 1% FBS. One hour prior to the assay, this media was removed, cells were rinsed twice with PBS, and media was replaced with DMEM containing 0.1% FBS. FLS (2.6 × 10^4 ^cells/well in DMEM + 0.1% FBS) with or without mitomycin C (MMC; 10 μg/ml; Sigma, St Louis, MO, USA) were added to the bottom wells of a 48-well microchemotaxis chamber (Neuroprobe, Gaithersburg, MD, USA). The chambers were inverted and incubated for 2 h (3 h when MMC was present) in a 5% CO_2 _atmosphere at 37°C, allowing for cell attachment to the membrane. Upon righting the chambers, dilutions of synovial fluids collected from patients with RA (or control agents) were added to the top wells and the chambers were incubated overnight at 37°C. PBS served as a negative control, whereas recombinant human basic fibroblast growth factor (bFGF; R&D Systems, Minneapolis, MN, USA) served as a positive control. The next morning, non-migrated cells were detached with a cotton swab, membranes were removed, fixed in methanol, and stained with Diff-Quik (Dade Behring, Deerfield, IL, USA). Checkerboard analysis was performed in a similar manner, except that the concentrations of RA SF were varied in the upper and lower chambers. Dilutions of RA SFs (1:100, 1:75 or 1:50) were added to the cell suspension in the bottom wells as well as on the opposite side of the membrane, when appropriate. Each condition was analyzed in quadruplicate and migrated cells from membranes mounted on glass slides were quantified in three representative high power fields. Quantification of high powered fields was accomplished by analyzing photographs of chemotaxis spots taken with a Nikon Coolpix E5000 (5.0 megapixel) camera mounted on a Nikon Eclipse TS100 inverted microscope. Chemotaxis data appeared normally distributed based on examination of histogram plots, and statistical analysis was performed using a Student's *t*-test.

To assure that local proliferation on the membrane was not occurring, WT and p21^(-/-) ^FLS were treated with identical conditions as used for chemotaxis assays described above. Cells were trypsinized, counted using trypan blue, plated at 2.6 × 10^4 ^cells/well into two separate chemotaxis chambers, and inverted to allow cell adherence to the membrane. FLS from one chamber were fixed with MeOH exactly 2 h after plating, allowing sufficient time for cell attachment but not enough time for proliferation. Counts from this chamber allowed an exact determination of the number of cells plated for each group. WT and p21^(-/-) ^FLS in the second chamber were plated with or without 10 μg/ml MMC. After 2 h, the second chamber was righted and PBS was added to the top side of all wells. After an 18 h time period, the time allowed for migration in all chemotaxis experiments, the membrane was removed and the WT and p21^(-/-) ^FLS were fixed with MeOH and counted.

### Restoration of p21 expression

To restore p21 expression, p21^(-/-) ^FLS were infected with an adenovirus overexpressing p21 (Adp21) and compared to mock infected WT FLS, mock infected p21^(-/-) ^FLS or p21^(-/-) ^FLS infected with an adenovirus that produces an irrelevant bacterial protein (β-galactosidase; AdlacZ). Initially, FLS infected with Adp21 and AdlacZ were subjected to β-galactosidase staining to estimate a concentration that resulted in infection of nearly 100% of cells (1 × 10^10 ^particles/1 × 10^5 ^cells; data not shown). All infections were completed by incubation with virus for 4 h in DMEM + 5% FBS. Mock infected WT and p21^(-/-) ^fibroblasts were incubated with the same media in the absence of virus. After incubation, cells were washed three times with PBS and full growth media was replaced for 4 h. Cells were incubated overnight in a 1:10 dilution of the full growth media. Chemotaxis assays were performed as described above the following day.

## Results

### p21^(-/-) ^FLS exhibit a faster growth rate than WT FLS, while both cell types migrate in a dose-dependent manner to RA SFs

**Figure 1:**
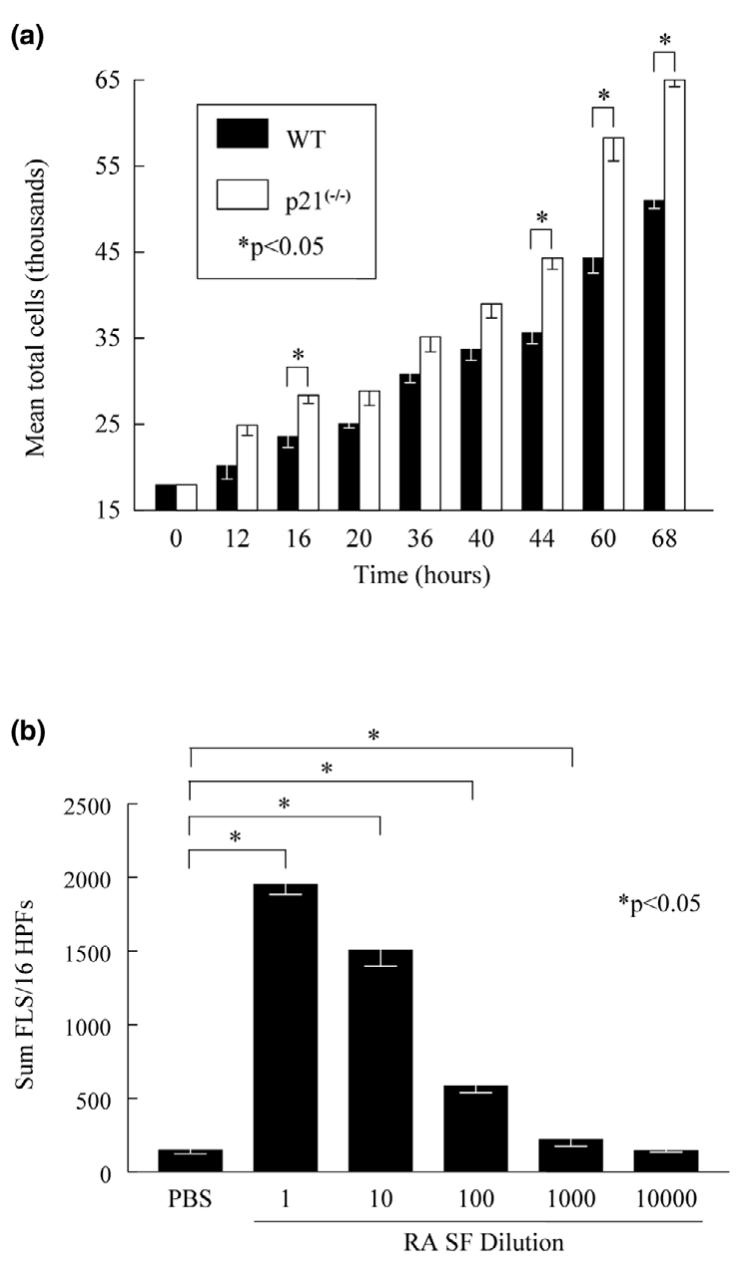
p21^(-/-) ^fibroblast-like synoviocytes (FLS) exhibit a faster growth rate than wild-type (WT) FLS, while both cell types migrate in a dose-dependent manner to rheumatoid arthritis (RA) synovial fluids (SFs). **(a) **Equal numbers of WT or p21^(-/-) ^FLS were plated into a 24-well plate and allowed to grow. At various time points, cells were removed and quantified as described in the Materials and methods section. Bars represent the mean of triplicate wells ± standard error (SE). An asterisk indicates a statistically significant difference. **(b) **WT FLS were tested for their ability to migrate to different dilutions of RA SF. The sum of counts from three high power fields (HPFs) was determined, and bars represent the mean of those sums from quadruplicate wells ± SE. An asterisk indicates a statistically significant increase in chemotaxis relative to background migration to PBS.

Little is known about whether the reduced p21 expression exhibited in RA FLS may be related to alterations in FLS migration. To address this issue, we isolated FLS from knee synovium of WT and p21^(-/-) ^mice. We first assessed whether FLS from p21^(-/-) ^mice possessed proliferative characteristics, as was expected from the loss of a cell cycle inhibitor. Figure 1a demonstrates that FLS isolated from mice lacking p21 do proliferate quicker than FLS isolated from WT mice. At time zero, equal numbers of WT and p21^(-/-) ^FLS were plated into 24-well plates. At various time points, cell number was determined and by 44 h after plating the cells, more p21^(-/-) ^FLS were consistently present relative to WT FLS (Figure 1a; p < 0.05). Next, we determined whether mouse FLS would migrate in response to human RA SF. We chose RA SF as a chemoattractant for these studies because of its relevance to arthritis and because these fluids are known to possess a combination of biologically relevant chemoattractants at levels that are sufficient to induce migration of other cell types [28]. We found that WT (Figure 1b) and p21^(-/-) ^(data not shown) FLS migrate to RA SF in a dose-dependent manner. Even RA SF dilutions of 1:1000 from some patients could significantly increase FLS migration when compared to background migration represented by PBS (p < 0.05).

### p21^(-/-) ^FLS migrate more than WT FLS in response to RA SF

**Figure 2:**
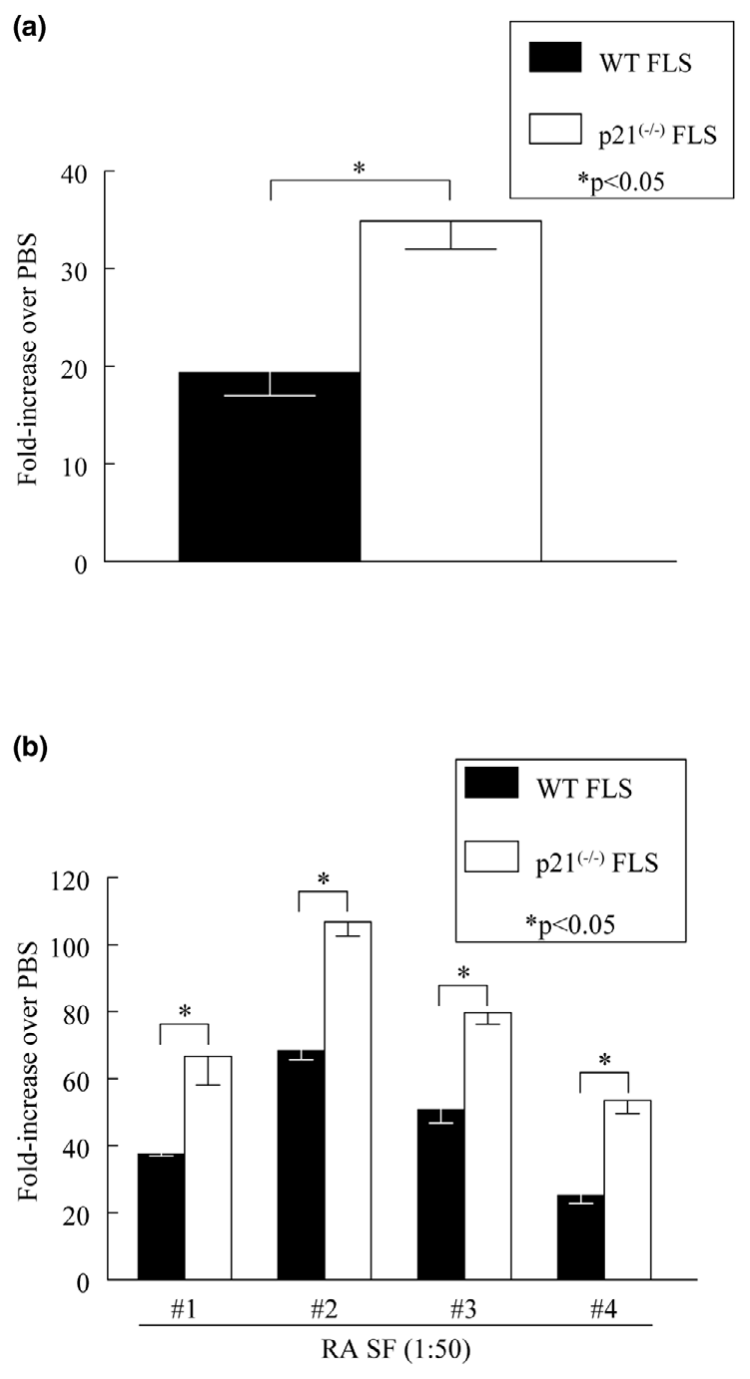
p21^(-/-) ^fibroblast-like synoviocytes (FLS) migrate more than wild-type (WT) FLS in response to rheumatoid arthritis (RA) synovial fluid (SF). WT and p21^(-/-) ^FLS were introduced to gradients of RA SFs (diluted in PBS) to induce migration. Cell counts are expressed as fold-increase over background migration to PBS (negative control). **(a) **Combined chemotaxis data analyzed from four separate patients in three independent experiments demonstrates significantly more migration by the p21^(-/-) ^FLS compared with WT FLS (p < 0.05). **(b) **Representative data from 1 of 3 independent chemotaxis assays performed using RA SF diluted 1:50 from 4 separate patients randomly designated #1 to #4. p21^(-/-) ^FLS migrated significantly (indicated by an asterisk) more than WT cells in response to all four RA SFs tested.

To directly compare migration of WT and p21^(-/-) ^FLS in response to RA SF, we employed 48-well microchemotaxis chambers and determined cell motility towards dilute RA SFs. Cell counts were normalized to their background migration by representing the data as fold-increase over PBS. Figure 2a shows combined chemotaxis data analyzed from four separate patients in three independent experiments and demonstrates significantly more migration by the p21^(-/-) ^FLS when compared with WT FLS (p < 0.05). A representative chemotaxis experiment is shown in Figure 2b, where RA SF was diluted 1:50 from 4 separate patients randomly designated #1 to #4. For this assay, the PBS counts used for normalization purposes were 6 for the WT FLS and 10 for the p21^(-/-) ^FLS. The data shown are representative of three independent experiments, which consistently suggest that p21^(-/-) ^FLS exhibit significantly enhanced migration towards RA SFs (p < 0.05).

### Migratory differences between WT and p21^(-/-) ^FLS are a mixture of chemotaxis and chemokinesis

To examine whether the increased migration of p21^(-/-) ^FLS may simply be the result of a non-specific enhancement of chemokinesis, we performed several checkerboard assays. A representative assay (Table 1) suggests that despite a minimal increase in random migration, there is also a large increase in specific chemotaxis when the cells are exposed to a greater concentration of RA SF on the opposite side of the membrane. We have previously reported a similar mixture of chemotaxis and chemokinesis [29]. These findings suggest that loss of p21 in FLS may enhance the cells ability to migrate towards the proinflammatory constituents of the RA joint.

### Enhanced migration of p21^(-/-) ^FLS to RA SF is independent of cell cycle regulation

**Figure 3:**
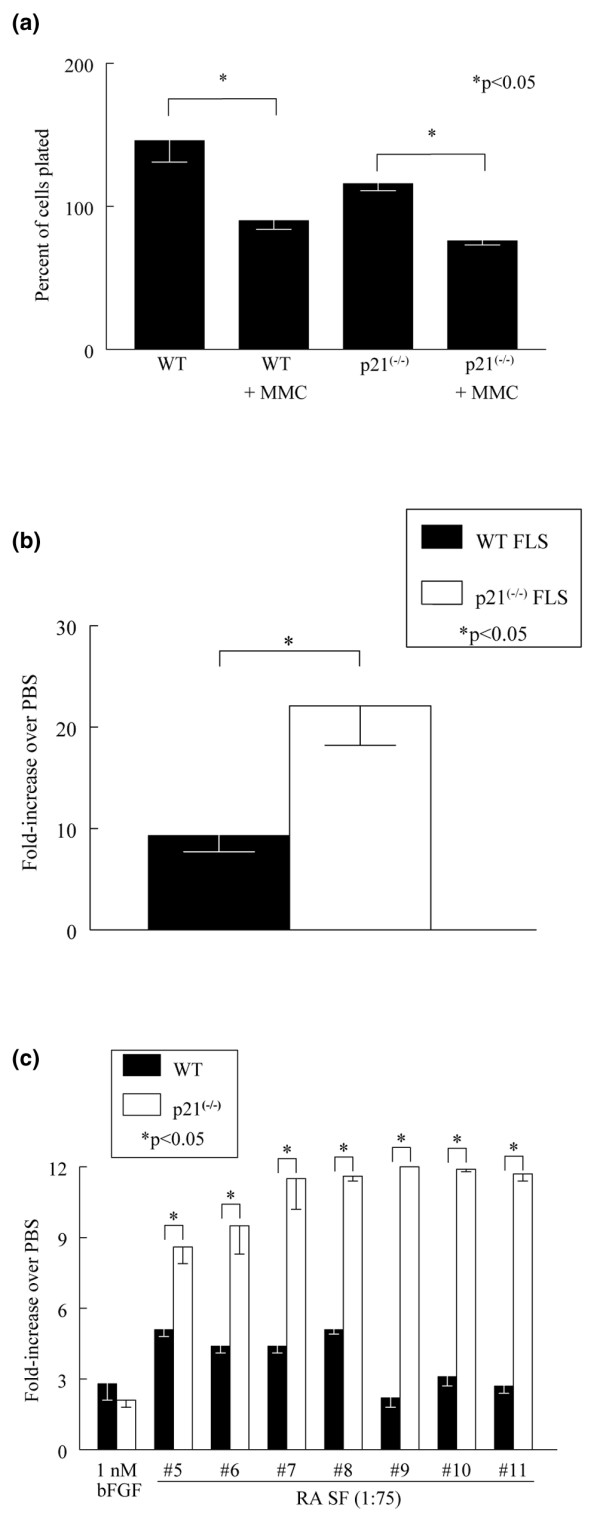
Enhanced migration of p21^(-/-) ^fibroblast-like synoviocytes (FLS) to rheumatoid arthritis (RA) synovial fluid (SF) is independent of cell cycle regulation. **(a) **Wild-type (WT) and p21^(-/-) ^FLS were treated identically to the chemotaxis assay in the presence and absence of mitomycin C (MMC) to determine whether the MMC conditions applied allowed for any proliferation of FLS. Cell counts are expressed as the percentage of cells plated, which was determined by counting the number of cells present after 2 h. The asterisk indicates a statistically significant difference between the groups, as determined from three identical experiments. **(b) **WT and p21^(-/-) ^FLS were allowed to adhere to the bottom side of the membrane with the chamber inverted in the presence of 10 μg/ml MMC. Subsequently, cells were introduced to gradients of RA SFs diluted 1:75 to induce migration. Cell counts are expressed as fold-increase over background migration to PBS (negative control). An asterisk indicates a statistically significant difference. Combined chemotaxis data analyzed from seven separate patients in nine independent experiments demonstrates significantly more migration by the p21^(-/-) ^FLS compared with WT FLS (p < 0.05). **(c) **A representative chemotaxis assay of 9 independent assays examining RA SF (1:75 dilution) from 7 separate patients randomly designated #5 to #11. bFGF, basic fibroblast growth factor.

To examine whether the enhanced migration could be explained by differences in proliferation, we performed chemotaxis assays using cells that were pretreated with MMC to stop cell division. To assure that local proliferation on the membrane was not occurring in the presence of MMC, we determined the number of WT and p21^(-/-) ^FLS in the presence and absence of MMC at the end of the assay and compared it with the number of cells plated at the start of the assay. Figure 3a shows that although FLS proliferation did appear to occur during the 18 h period, cells treated with MMC were at or below the number of cells plated. The number of WT FLS treated with MMC was significantly below the number of WT FLS that were not MMC treated (p < 0.05). Similarly, the number of p21^(-/-) ^FLS treated with MMC was significantly below the number of p21^(-/-) ^FLS that were not MMC treated (p < 0.05). In contrast, there were no significant differences between WT and p21^(-/-) ^FLS that were or were not MMC treated. This suggests that MMC completely halted proliferation and that any demonstrable changes in chemotaxis in the presence of MMC are not due to proliferation on the membrane.

Figure 3b demonstrates that when data are combined from p21^(-/-) ^and WT FLS chemotaxis assays using seven separate RA SFs in nine independent experiments, there is significantly more migration by the p21^(-/-) ^FLS compared with WT FLS (p < 0.05). Figure 3c shows a representative chemotaxis assay that employed RA SFs from seven different patients. For this assay, the PBS counts used for normalization purposes were 61 for the WT FLS and 45 for the p21^(-/-) ^FLS. It should be noted that the counts in chemotaxis assays using mouse FLS isolated on different dates occasionally varied greatly between experiments (background migration as well as induced migration), although the trend was always identical, such that the p21^(-/-) ^FLS migrated significantly more than the WT FLS. Therefore, in the presence of MMC, a cell division inhibitor, we still demonstrate that loss of p21 confers an increased migratory behavior on FLS when compared with WT cells (p < 0.05). Of note in Figure 3c, we included migration towards 1 nM bFGF, a factor known to induce migration of fibroblasts. Indeed, bFGF induced a two- to three-fold increase in cell migration over PBS values, although this migration displayed no significant differences between WT and p21^(-/-) ^FLS.

### WT and p21^(-/-) ^FLS exhibit no differences in migration to bFGF

**Figure 4:**
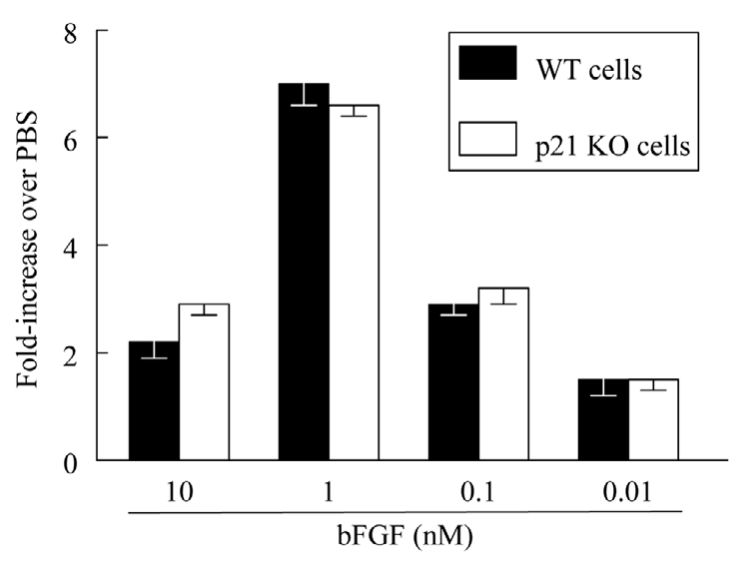
Wild-type (WT) and p21^(-/-) ^fibroblast-like synoviocytes (FLS) exhibit no differences in migration when basic fibroblast growth factor (bFGF) is used as the chemoattractant. Migratory differences between WT and p21^(-/-) ^FLS were assessed using various concentrations of bFGF as the chemoattractant. Cell counts are expressed as fold-increase over background migration to PBS (negative control). Results are representative of seven independent assays. KO, knockout.

To begin to assess whether the enhanced migration may exhibit specificity with regards to the chemoattractant used, we performed several chemotaxis assays using bFGF as the chemoattractant. Figure 4 shows that when we used bFGF as a sole chemoattractant (representative of seven independent chemotaxis assays), we did not see consistent differences between migration of p21^(-/-) ^and WT fibroblasts. Therefore, the enhanced migration may be specific for another chemoattractant, or a combination of chemoattractants found in the RA SF.

### Restoration of p21 in p21^(-/-) ^FLS significantly reduces excessive migration to RA SF

**Figure 5:**
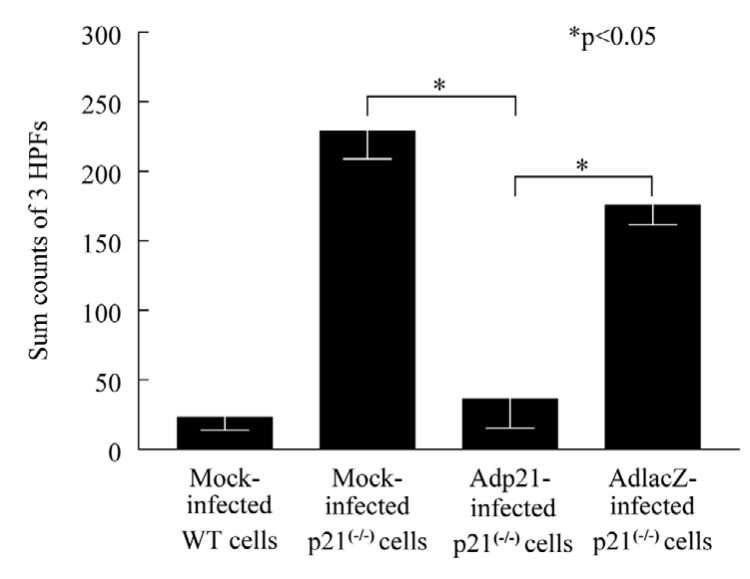
Restoration of p21 in p21^(-/-) ^fibroblast-like synoviocytes (FLS) significantly reduces excessive migration to rheumatoid arthritis (RA) synovial fluid (SF). Cells were infected as described and used for chemotaxis the following day, when nearly all FLS were estimated to express their recipient transgene. Three high powered fields (HPFs) of migrated cells were counted and their sum was determined for each well. Bars represent the mean of quadruplicate wells ± standard error (SE) and an asterisk indicates a statistically significant difference. These data are representative of three independent assays. AdlacZ, adenovirus expressing β-galactosidase; Adp21, adenovirus expressing p21; WT, wild type.

We next aimed to use an adenovirus to increase p21 and establish whether reconstituting p21^(-/-) ^FLS with p21 would reduce their excessive migration. First, to demonstrate that our adenoviral construct produced functional p21, we infected p21^(-/-) ^FLS and compared the growth rate with that of p21^(-/-) ^FLS infected with an adenovirus producing green fluorescent protein. Growth of FLS expressing p21 was significantly inhibited compared with FLS expressing green fluorescent protein (p < 0.05; data not shown). Next, to establish whether p21 is solely responsible for the enhanced migratory characteristics, we reconstituted p21^(-/-) ^FLS with p21 and compared chemotaxis to cells infected with AdlacZ. Figure 5 demonstrates that the migration of p21^(-/-) ^FLS infected with Adp21 was comparable to WT FLS that were mock-infected. This migration was significantly below mock-infected p21^(-/-) ^FLS or those infected with an adenovirus producing an irrelevant bacterial protein, β-galactosidase.

## Discussion

Expression of p21 in RA synovial tissue is significantly decreased when compared with the same tissue from osteoarthritis patients. Expression of p21 inversely correlates with thickness of the RA synovial lining [20]. Similarly, FLS isolated from RA patients express significantly less p21 than FLS isolated from osteoarthritis patients. Little is known, however, about whether the reduced p21 expression found in RA FLS may be related to alterations in FLS migration. As a constituent of the synovial pannus in RA, FLS have long been identified as key players in the aggressive invasion of cartilage and bone, contributing to joint damage [30]. To address the issue of whether the lack of p21 in RA FLS may alter the migratory properties of these cells, we isolated FLS from knee synovium of WT and p21^(-/-) ^mice. We first assessed whether FLS from p21^(-/-) ^mice possessed proliferative characteristics, as was expected from the loss of a cell cycle inhibitor. Figure 1a demonstrates that FLS isolated from mice lacking the cell cycle inhibitor p21 do indeed multiply quicker than FLS isolated from WT mice. Next, we demonstrated that mouse FLS migrate in a dose-dependent manner to human RA SF. We chose RA SF as a chemoattractant for these studies because of its relevance to arthritis, where RA FLS express significantly less p21, in addition to the fact that these fluids are known to possess a combination of biologically relevant chemoattractants at levels that are sufficient to induce migration [28].

A direct comparison of WT and p21^(-/-) ^FLS migration to RA SF suggests that p21^(-/-) ^FLS exhibit significantly enhanced migration (p < 0.05). Our checkerboard assays (Table 1) suggest that this may be a mixed combination of mainly chemotaxis with some chemokinesis. Use of MMC to inhibit the cell cycle completely halted proliferation of both cell types on the chemotaxis membrane. While Figure 1a clearly demonstrates that p21^(-/-) ^FLS proliferate quicker than WT FLS, the non-MMC treated FLS in Figure 3a did not show this trend, most likely due to significant differences in the design of these two experiments. For example, the FLS used for Figure 1 had access to 10% serum continuously while growing on non-coated plates, while FLS used for Figure 3a had 1% serum for 18 h and then 0.1% serum for the last hour before being plated onto a gelatin coated membrane. It is possible that these differences in experimental design masked the growth difference between the two cell types in this shortened time frame. Also, a comparison of the representative assay shown in Figure 2b with that shown in Figure 3c should not suggest that migration in the absence of MMC was greater than migration in the presence of MMC. The increased migration relative to PBS varied greatly when comparing FLS that were isolated from pooled mouse knees on different dates. However, the trend was always consistent, such that the FLS from p21^(-/-) ^mice migrated more than those from WT mice. Overall, chemotaxis assays performed in the presence of a cell cycle inhibitor suggest that the changes noted are not the result of enhanced proliferation by migrating cells. Moreover, reconstitution of p21 into p21^(-/-) ^FLS is sufficient to significantly reduce migration of these cells, suggesting that the loss of p21 in FLS may enhance their ability to migrate towards the proinflammatory constituents of the RA joint.

Recently, Besson and coworkers [17] demonstrated that p27, a cell cycle inhibitor with high homology to p21, also plays a role in regulating cell migration. While our data suggest that FLS lacking p21 have enhanced migratory ability, this recent study reports that murine embryonic fibroblasts lacking p27 exhibit a dramatic decrease in cell motility, the exact opposite response, in a cell-cycle independent manner. p21^(-/-) ^embryonic fibroblasts were also examined in this study, but were determined to not exhibit differences in migration when compared with WT cells [17]. There are several major differences between the designs of our studies, which likely account for the novel results that we are reporting with p21. For example, our study included p21^(-/-) ^fibroblasts that were isolated from synovium obtained from 5–8 week old mice, whereas the previous study employed fibroblasts derived from an embryo. The previous study examined cell migration by wounding of a confluent monolayer of cells, whereas our study examined specific directed chemotaxis and chemokinesis. An additional key difference is that our study used RA SF as the chemoattractant to assess whether the multiple biological constituents of these disease-related fluids may display differences in chemoattraction between p21^(-/-) ^and WT fibroblasts. In fact, when we utilized bFGF as a sole chemoattractant in seven independent chemotaxis assays, we do not see consistent differences between migration of p21^(-/-) ^and WT fibroblasts, which is in-line with the previously published findings where p21^(-/-) ^assays were performed in the presence of growth serum [17].

Previous studies in vascular smooth muscle cells (VSMCs) involving the homeobox transcription factor growth-arrest specific homeobox (Gax) have also established a tie between p21 and cell migration [31]. Overexpression of Gax in VSMCs has an antiproliferative effect induced by the upregulation of p21 [32]. Moreover, transduction of Gax cDNA inhibits VSMC migration to a variety of chemoattractants, an effect that is lost when attempted in p21^(-/-) ^VSMCs [31]. Upon restoring p21^(-/-) ^cells with exogenous p21, transduction of Gax cDNA once again inhibits VSMC migration. Thus, increasing Gax upregulates p21 and inhibits VSMC migration. This appears consistent with our studies, in which a loss of p21 in FLS results in excessive migration and reconstituting p21 is capable of reducing the exuberant migration to RA SF. Overexpressing p21 alone in WT VSMCs did not influence cell migration. In our hands, similarly, infecting WT FLS with a high titer of Adp21 did not inhibit cell migration to RA SF (data not shown).

FLS locomotion can be regarded as an important pathogenic mechanism contributing to the invasion of cartilage and bone in RA. Grafting of RA FLS alone to SCID mice, in the absence of a functional immune system, results in chronic arthritis, underscoring the potential key role of these cells in driving disease [19,33]. This is an area of intense research interest, where invasiveness of RA FLS *in vitro *has recently been associated with the rate of joint destruction *in vivo *[34]. Invasiveness of RA FLS varies on a patient-by-patient basis [34], and these same RA FLS have been shown to express significantly lowered levels of p21 [20]. We demonstrate that the loss of p21 in FLS results in a significant increase in migration towards the combination of biologically relevant chemoattractants found in multiple RA SFs. In addition, this effect is independent of the cell cycle activity of p21, and restoring p21 can reconstitute migration to levels comparable to WT cells. These findings, in combination with previous studies performed in VSMCs, suggest that p21 may be a key regulator of cellular migration, with particular importance to RA.

## Conclusion

We previously demonstrated that RA FLS exhibit decreased expression of p21, and now observe that a lack of p21 may contribute to excessive migration of FLS. These data suggest that p21 plays a novel role in normal FLS in repression of migration. Further, a lack of p21 expression, as occurs in RA FLS, may contribute to excessive invasion towards the biological chemoattractants found in RA SF.

## Abbreviations

AdlacZ = adenovirus expressing β-galactosidase; Adp21 = adenovirus expressing p21; bFGF = basic fibroblast growth factor; cdk = cyclin-dependent kinase; DMEM = Dulbecco's modified Eagle's medium; FBS = fetal bovine serum; FLS = fibroblast-like synoviocytes; Gax = growth-arrest specific homeobox; MMC = mitomycin C; PBS = phosphate-buffered saline; RA = rheumatoid arthritis; SF = synovial fluid; VSMC = vascular smooth muscle cell; WT = wild-type.

## Competing interests

The authors declare that they have no competing interests.

## Authors' contributions

JMW designed and developed all aspects of the study, performed chemotaxis experiments, drafted the manuscript, and addressed reviewers concerns. KK, DJS, NGS, and MSR each contributed a significant number of chemotaxis assays, worked with adenoviruses, and participated in growth and maintenance of FLS. DJP performed the majority of chemotaxis counting, while MVV participated in chemotaxis counting as well as in the design of experiments and interpretation of data. JCS performed all animal work and isolation of FLS cultures from mice. HP conceived of the study and participated in its design and coordination. All authors read and approved the final manuscript.

## Figures and Tables

**Table 1:** Migratory differences between wild-type and p21^(-/-) ^fibroblast-like synoviocytes are due to altered chemotaxis and chemokinesis

	RA SF dilution across membrane			
RA SF dilution in lower wells	None	1:100	1:75	1:50

None	3 ± 0.9	142 ± 9	156 ± 11	194 ± 9
1:100	16 ± 3	133 ± 8	122 ± 5	185 ± 13
1:75	18 ± 2	92 ± 3	143 ± 7	209 ± 7
1:50	12 ± 2	116 ± 8	100 ± 4	130 ± 9

Checkerboard assay results of p21^(-/-) ^fibroblast-like synoviocytes (FLS) migration are presented such that dilutions of rheumatoid arthritis (RA) synovial fluids (SFs), which were included with the cells (in the lower wells), are displayed down the left hand column. Dilutions of RA SFs across the membrane from the cells are displayed in the top row. Numbers represent the sum of counts from three high power fields determined from quadruplicate wells ± standard error (SE). These data are representative of three independent checkerboard assays.
